# Evaluating the effectiveness of the education program developed for the empowerment of new graduate nurses: A randomized controlled trial

**DOI:** 10.1111/jnu.13041

**Published:** 2024-12-10

**Authors:** Seda Sarıköse, Sevilay Şenol Çelik

**Affiliations:** ^1^ Koç University School of Nursing Istanbul Turkey

**Keywords:** education program, flipped classroom, new graduate nurse, psychological empowerment, randomized controlled trial, structural empowerment

## Abstract

**Purpose:**

To evaluate the effectiveness of the education program developed based on the structural empowerment (SE) and psychological empowerment (PE) theories and flipped classroom model for the empowerment of new graduate nurses (NGNs).

**Design:**

Single‐center, parallel‐group, randomized controlled trial.

**Methods:**

The study was conducted between June 2021 and September 2023 in two phases: developing the education program to empower NGNs and evaluating its effectiveness. An education program consisting of two parts, online and face‐to‐face, was developed. The online part consists of eight modules implemented for two weeks. The face‐to‐face part was implemented for two days and included the in‐class activities. NGNs were randomly assigned to the intervention group (*n*: 32) and control group (*n*: 32). An education program was applied to the intervention group, whereas the control group continued their routine orientation program. A range of outcome measures of SE, PE, and education programs' effectiveness were evaluated. Data were analyzed using descriptive, chi‐squared, and *t*‐tests.

**Results:**

The study determined that the intervention and control groups showed homogeneous distribution in the pretest. A statistically significant difference was identified between the intervention and control groups regarding the mean scores of PE and SE three months following the implementation of the education program, and the total mean score of the intervention group was higher.

**Conclusion:**

The education program developed to empower NGNs was a highly effective intervention in increasing nurses' perceptions of SE and PE. There is a need to carry out studies and activities to disseminate this program.

**Clinical Relevance:**

The findings of this study will guide educators, researchers, and administrators in future strategies and innovative programs for empowering NGNs.

## INTRODUCTION

New graduate nurses (NGNs) are essential to healthcare systems, contributing to the delivery of high‐quality nursing care and ensuring patient safety. However, due to their limited clinical experience in patient care management and unfamiliarity with institutional protocols and procedures, NGNs face significant challenges in adapting to the demands of the healthcare environment (Lyu et al., 2024). NGNs require time to develop their skills and gain experience in the workplace before becoming proficient registered nurses. However, the transition from NGNs to experienced nurses can be challenging (Mirza et al., 2019). Previous research has indicated that the reality shock faced by NGNs can result in both individual and organizational challenges, including burnout, reduced performance, decreased job satisfaction, medical errors compromising patient safety, and higher rates of absenteeism, turnover, and attrition from both the job and the nursing profession (Flinkman & Salanterä, 2015; Kuokkanen et al., 2016). Findings indicate that 27.3% of NGNs leave their profession within the first year of employment (Nursing Solutions Inc., 2021). This rapid turnover significantly impacts both patient care and financial aspects. Notably, a mere 1% change in nurse turnover could result in hospitals either saving or spending approximately $379,500 (Joseph et al., 2022). Healthcare managers find the increasing costs of the adaptation process challenging for the organization (Bowblis & Roberts, 2020). For this reason, to prevent increasing costs, to provide quality and safe healthcare services, and to retain NGNs, international healthcare institutions and organizations, and scientists are aiming to develop strategies to facilitate the transition and empowerment of NGNs to the role of professional nurses (Bowblis & Roberts, 2020).

Empowerment is defined as situations and working environments that enable employees to increase their motivation toward their jobs, roles, and responsibilities and their willingness to take action by using their expert power in line with organizational values (Vu, 2020). Empowerment in nursing is defined as enhancing the quality of patient care through the improvement of job satisfaction and productivity, achieved by the allocation of organizational resources and support (Al‐Dweik et al., 2016; Kuokkanen et al., 2016). Empowerment of nurses can be achieved by developing the professional identities of nursing students starting from the education and training process (Al‐Dweik et al., 2016). For this reason, some educational institutions adopt an integrated education system in which theoretical and practical education is given in coordination to empower nursing students and include high‐tech simulation and virtual reality practices and critical thinking, stress and time management, leadership, ethics, and effective communication in their curricula to improve students' professional skills (Al‐Dweik et al., 2016; Saab et al., 2021). Today, not every institution may have sufficient physical structure and vision to empower nursing students (Albasal et al., 2022). In this context, the empowerment of nurses becomes an issue that institutions should address after graduation.

## BACKGROUND

### Theoretical foundation for the study

Researchers delve into the study of empowerment through structural and psychological dimensions. Kanter's (1977) framework of structural empowerment and Spreitzer's (1995) model of psychological empowerment serve as foundational frameworks in national and international studies focused on empowering nurses. The main objective of the SE theory developed by Kanter (1977) is to create healthy working environments that provide employees at all levels to take an active role in organizational decision‐making. The SE theory in nursing is characterized by the distribution of organizational resources and authority to enhance patient care quality, boost productivity, and enhance satisfaction (Wafa'a et al., 2020). SE theory is examined in six subdimensions, defined as access to opportunities, information, resources and support, and formal and informal power (Kanter, 1977). Employees who cannot access information, support, and resources may be unable to do their jobs properly and feel hopeless, powerless, and dissatisfied (Laschinger et al., 2001). The studies found that organizations that engage in activities based on the SE theory reward nurses by increasing their competence and skill levels, and with this approach, nurses' perceptions of empowerment increase (Kuokkanen et al., 2016; Özbaş & Tel, 2016; Yang et al., 2014).

PE theory, developed by Spreitzer (1995), consists of four subdimensions: meaning, autonomy, competence, and influence. PE theory aims to help employees feel competent and effective in their professions and work by finding their duties meaningful. Also, the empowerment programs need to have practices that increase the resilience of NGNs, the meaning of their profession, their professional competence, influence, and autonomy through professional knowledge and skills development. Many researchers recommend PE theory to provide NGNs with these competencies (Im et al., 2016; Özbaş & Tel, 2016; Yang et al., 2014). Studies have mentioned the relevance of SE and PE in providing NGNs with a deep commitment to professional values and a clear sense of identity (Dahinten et al., 2014; Im et al., 2016; Özbaş & Tel, 2016; Yang et al., 2014). Researchers recommend developing transition and education programs based on these theories to empower NGNs (Kuokkanen et al., 2016; Murray et al., 2020; Sarıköse & Çelik, 2024). Thus, the theoretical framework of this study and the empowerment program for NGNs is based on SE and PE theories.

### Literature supporting the use of the theory from previous research

There are limited empowerment‐based programs besides the transition programs prepared for NGNs. It is known that empowerment programs developed for nurses mainly include leadership and management issues and are aimed at nurse managers or staff nurses (Bard et al., 2022). Studies showed that these programs implemented for NGNs increase their leadership skills, perceptions of SE and PE, levels of organizational commitment, control over nursing practices, and perceptions of patient safety, as well as reduce burnout levels and turnover rate (Dahinten et al., 2014; Im et al., 2016; Özbaş & Tel, 2016; Yang et al., 2014). The empowerment levels of NGNs working in organizations that provide their nurses with orientation and training programs, etc., were higher (Sarıköse & Çelik, 2024). Therefore, researchers are aiming to develop empowerment programs for NGNs. Bard et al. (2022) developed an online empowerment program for nurses directly involved in patient care processes, including new graduates. Im et al. (2016) developed an innovative empowerment program called “Huddling” to empower NGNs in Korea and increase their organizational commitment and resilience.

According to studies, it has been determined that education programs developed for NGNs are based on various educational methods (Dahinten et al., 2014; Im et al., 2016; Özbaş & Tel, 2016; Yang et al., 2014). The millennial generation, including NGNs, is known to be more resistant to traditional lecture‐based educational processes (Cha & Kim, 2020). Accordingly, the flipped classroom model is considered an innovative and effective learning approach that meets the educational needs of NGNs (Cha & Kim, 2020; Chen & Chen, 2014). The essential principle of the flipped classroom model is that learners come to class prepared for in‐class activities, discussions, and interactions (Chen & Chen, 2014). This model provides a more dynamic and interactive learning environment compared to traditional education. It transforms the pedagogical approach into an interactive learning environment, offers individual learning opportunities by transferring course content to a digital format, and allows educators to guide learners with creative and innovative educational methods (Chen & Chen, 2014). Considering the conditions of institutions and organizations providing nursing education and healthcare services, every nurse cannot reach these programs prepared for their empowerment under equal conditions (Kuokkanen et al., 2016). In this context, there is a need for an effective and modern education program for the empowerment of NGNs. This study aims to evaluate the effectiveness of the education program developed based on the SE and PE theories to empower NGNs.

### Research hypothesis

H1: The SE levels of the NGNs in the intervention group who participated in the theory‐based education program and flipped classroom model will be higher than that of the control group, who participated in the routine orientation program.

H2: The PE levels of the NGNs in the intervention group who participated in the theory‐based education program and flipped classroom model will be higher than that of the control group, who participated in the routine orientation program.

## METHODS

### Design

The study was designed as a single‐center, parallel‐group, randomized controlled trial (RCT) to evaluate the effectiveness of the education program developed based on the flipped classroom model, SE, and PE theories to empower NGNs. This study adhered to the Consolidated Standards of Reporting Trials‐CONSORT guidelines for evaluating nonpharmacological interventions (Data S1), and the trial protocol was registered at clinicaltrials.gov (ID no: NCT05597618).

### Participants and setting

The study sample consisted of NGNs working at the private university hospital with bachelor's levels of nursing education. All the NGNs included in the study completed 56 hour of face‐to‐face routine orientation program provided by their institutions. This orientation program consists of training on hospital services, effective communication and teamwork, patient safety, and fundamental nursing skills. An educational program developed specifically for the empowerment of NGNs was implemented in the intervention group. However, the control group did not receive any additional interventions and only participated in the routine orientation program provided by their institutions.

**FIGURE 1:**
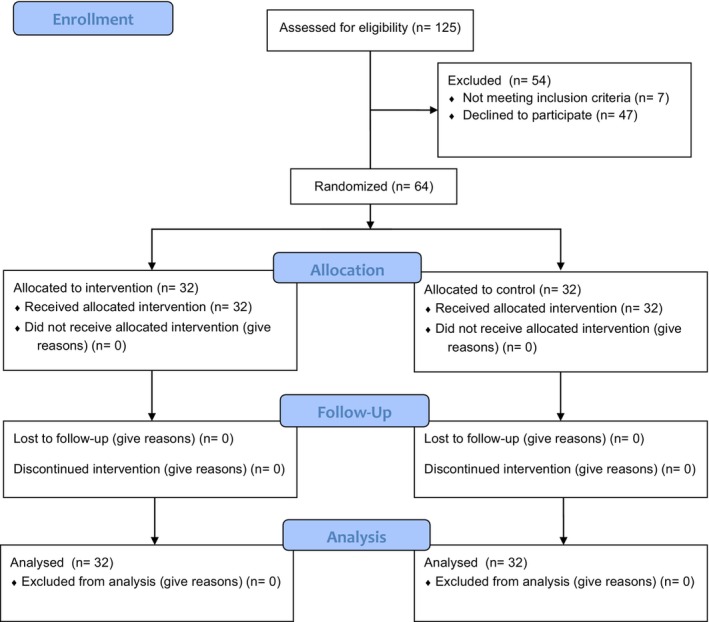
Consort flow chart.

Studies suggest that transition programs for empowering NGNs should be within the first 12 months of their professional experience (Andregård & Jangland, 2015). For this reason, NGNs at the bachelor's degree level with a total working experience of <12 months were included in the sample. In calculating the study's sample size, the average scale score used in a similar study (Im et al., 2016) was taken as a reference. Based on a power analysis conducted using G*Power 3.1.9.6 software, with 90% power, an effect size of 0.81, and a Type I error rate of 0.05, the study sample was determined to consist of 64 participants, with 32 assigned to the intervention group and 32 to the control group. Recruitment and enrolment of the participants are described in a trial flow diagram (Figure 1). Using a simple random numbers table, 64 participants were randomly chosen from the hospital's list of NGNs. After obtaining their consent and assessing the inclusion criteria, these 64 NGNs were randomly allocated to either the intervention or control group (Figure 1). A research assistant who was not involved in the study assigned these nurses to the intervention and control groups using the block randomization method (Research Randomizer). In the study, the blind technique was used in the randomization process and data evaluation phase. Throughout the assignment process, both the participants and the researcher were blinded to group allocation (intervention or control) until the commencement of the study. The random assignment was concealed using the closed envelope technique to ensure the integrity of the blinding process.

#### Inclusion criteria for participants in the RCT sample

- Total experience in the profession of 12 months or less.
- Minimum requirement of a bachelor's degree in nursing.

#### Exclusion criteria for participants in the RCT sample

- Total experience in the profession over 12 months.
- Quitting the job during the RCT.
- Having an educational background below the bachelor's degree level in nursing.

### Education program developed for the empowerment of NGNs

This program, developed based on SE and PE theories, consists of online and face‐to‐face education. In terms of meeting adult education needs and offering an innovative education model, the flipped classroom model based on Chen and Chen (2014), which explains the application principles of the flipped classroom model, was taken as a basis in the education program. This education program aims to provide NGNs with cognitive and affective learning skills, but it is not aimed at providing psychomotor skills. For this reason, the educational objectives and content of the research were prepared by considering Bloom's taxonomy (Bloom, 1956). The education contents and practices were prepared based on SE and PE theories, subdimensions of these theories, and the Ministry of Health's “Guideline on Essential Competencies in Nursing” in 2021 (Kanter, 1977; MoH, 2021; Spreitzer, 1995). The relationship between the contents of the education program and empowerment theories is given in Table 1. At the end of the six expert opinions, the education program was revised into its latest version. Five of these experts were academic faculty members specialized in nursing education, and one of them was an expert in education technologies.

**TABLE 1:** The relationship between structural and psychological empowerment theories and the contents of the education program.

Education contents	Structural empowerment						Psychological empowerment			
	Opportunities	Information	Resources	Support	Formal power	Informal power	Competence	Impact	Meaning	Autonomy
Empowerment	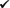	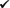	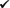	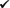	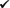	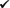	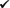	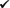	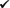	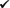
Competence in Nursing				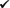	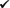	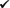	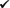	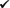		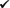
Problem‐Solving and Decision‐Making			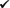	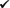		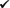				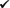
Time and Stress Management			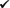	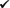					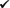	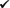
Nursing Legislation	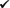	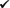					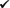			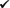
Ethics		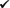							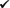	

#### Online education program

The online education consists of eight modules: “Introduction, Empowerment, Competence in Nursing (evidence‐based practices, effective communication, care management, professionalism, leadership, nursing care quality), Problem‐Solving and Decision Making, Time and Stress Management, Nursing Legislation, Ethics, and Finalizing” In the online education part, there were recorded educational videos, educational slides, and educational documents for each module, as well as online exercises consisting of five multiple‐choice questions. Through the system, achievement criteria were defined for the transitions between the modules. Participants were able to pass to the next module after successfully completing 80% of the multiple‐choice questions at the end of each module. Educational videos in online education modules take an average of 15 min. The educational videos were prepared by a total of six nursing faculty members who are experts in their fields. In addition, NGNs could write their feelings, thoughts, opinions, and suggestions about education or their questions to the educators through the forum section at the end of each module. Green screen technology was used to prepare educational videos (Data S2). This part was available on the Koç University Online Program platforms, which is mobile‐friendly.

#### Face‐to‐face education

This part was developed for two days in a classroom in the institution where the researchers worked. Face‐to‐face education started at 10 am and ended at 5 pm, and 10 hour of face‐to‐face education were completed. This part was also completed with the involvement of the educators in the online education part. The first day of education consisted of in‐class activities related to empowerment, competence in nursing, problem‐solving, and decision‐making. The second day of education consisted of in‐class activities on time and stress management, nursing legislation, and ethics. In‐class activities were prepared by taking the opinions of educators and experts, and small group studies, case discussions, brainstorming, art therapy and mindfulness techniques, and some digital tools, including Kahoot and Mentimeter, were used. The education evaluation methods consisted of question and answer, educator observations, educator feedback, and assignment evaluations.

### Instruments

#### Descriptive information questionnaire

This form was developed based on existing literature (Mansour et al., 2022; Mirza et al., 2019; Ortiz, 2016). The questionnaire gathers sociodemographic data (age, gender, marital status) and professional characteristics of NGNs, such as work experience, weekly working hours, shift type, and previous empowerment training. It includes a total of eight questions.

#### The Conditions for work effectiveness questionnaire (CWEQ‐II)

This scale was developed to assess four primary dimensions of workplace empowerment, specifically evaluating perceived access to opportunity, support, information, and resources, in alignment with Kanter's (1977) structural empowerment theory. This instrument was subsequently refined by Laschinger et al. (2001), who reported robust model fit indices for its factor structure, as indicated by *χ* ^2^ = 279, df = 129, CFI = 0.992, IFI = 0.992, and RMSEA = 0.054 in their confirmatory factor analysis. The Turkish version of the CWEQ‐II was validated and tested for reliability by Mortaş (2005), who conducted an exploratory factor analysis that revealed a six‐factor structure. First‐ and second‐order factor analyses demonstrated an adequate fit between the data and the theoretical model in the context of nursing. Mortaş (2005) further reported a Cronbach's alpha of 0.90 for the total scale, with subscale reliability coefficients ranging from 0.74 to 0.90, suggesting that the Turkish version possesses acceptable psychometric properties.

The CWEQ‐II comprises 19 items organized into six subscales: opportunity (e.g., the degree to which individuals can acquire new skills in their role), information (e.g., access to knowledge on the current state of the organization), support (e.g., availability of advice for problem‐solving), resources (e.g., sufficient time to fulfill job requirements), informal power (e.g., being consulted by colleagues), and formal power (e.g., recognition for innovative contributions). Each subscale, except for informal power, consists of three items. The Global Empowerment subscale, comprising two additional items, was integrated into the CWEQ‐II to ensure construct validity. This subscale was not included in the scale score. Laschinger et al. (2001) found a positive correlation between the Global Empowerment subscale and the total CWEQ‐II score (*r* = 0.56), reinforcing the scale's construct validity.

A 5‐point Likert‐type scale is derived in the CWEQ‐II, with separate scores computed for each subscale. An overall empowerment score can be derived by summing the scores across all subscales. Nurses' perceptions of empowerment are categorized based on total scores as low (6–13 points), moderate (14–22 points), or high (23–30 points). The Cronbach's alpha coefficient for the overall scale was 0.94 in this study, and 0.80, 0.81, 0.76, 0.83, 0.76, and 0.75 for the opportunity, information, support, resources, formal power, and informal power subscales, respectively. The GE subscale yielded a Cronbach's alpha of 0.82.

#### The Psychological Empowerment Scale (PES)

The Psychological Empowerment Scale, developed by Spreitzer (1995), assesses participants' perceptions of psychological empowerment. The scale includes four sub‐dimensions comprising 12 items. The first dimension of PES refers to the alignment between the demands of a job role and an individual's beliefs, values, and behaviors. The second dimension, competence, pertains to an individual's confidence in performing tasks proficiently. The third dimension, autonomy, highlights an individual's sense of autonomy in initiating and controlling their actions. The final dimension, impact, concerns how an individual can affect strategic, administrative, or operational outcomes in the workplace. The construct validity of the scale developed by Spreitzer (1995), the Psychological Empowerment Scale (PES), was assessed using second‐order confirmatory factor analysis (CFA). Laschinger et al. (2001) reported that the factor structure of this scale demonstrated acceptable fit indices, including *χ* ^2^ = 4.63, df = 1, CFI = 0.97, IFI = 0.97, and RMSEA = 0.14. In a study conducted by Uner and Turan (2010), exploratory factor analysis revealed a four‐factor solution across both models, with first‐ and second‐order CFA confirming a suitable fit between the data and the theoretical framework within nursing. The Cronbach's alpha values for the subscales ranged from 0.81 to 0.94, indicating satisfactory reliability for the Turkish version of the scale.

Uner and Turan's (2010) research further validated the PES in Turkish. This scale utilizes a 5‐point Likert‐type format, with the overall score determined by summing the responses to each item. The minimum score, 12, reflects a low level of perceived empowerment, whereas the maximum score, 84, indicates a high level. For the current study, the Cronbach's alpha for the total scale was 0.93, with the subscales reporting the following values: 0.76 for “competence,” 0.75 for “meaning,” 0.80 for “autonomy,” and 0.88 for “impact.”

#### Education evaluation form

This form was developed to evaluate the perceptions of NGNs in the intervention group regarding the educational program. The design of the form was guided by relevant literature (Chen & Chen, 2014; Phye et al., 2005). It was finalized after obtaining feedback from six experts specializing in nursing education and conducting a pilot study with five nurses who were not part of the main study sample. This form consists of two parts, each utilizing a 5‐point Likert scale. Scores approaching five indicate that the relevant statement is highly applicable to the education. In the first part, four items are designed to evaluate participants' opinions on the functioning of the face‐to‐face education component. These items assess the adequacy of the education period, the suitability of the education content, the appropriateness of the education method, and the adequacy of the environment in which the education is conducted. In the second part, four items are intended to evaluate participants' opinions regarding the functioning of the online education program. These items assess the adequacy of the duration of the educational videos, the appropriateness of the content of the education modules, the audio and visual quality of the educational videos, and the user‐friendliness of the online education program. Additionally, three items are aimed at evaluating participants' opinions regarding the benefits gained from the face‐to‐face and online education parts. These items assess the contribution to professional development, the contribution to the field of clinical practice, and the contribution to interest in the subject.

### Ethical considerations

Approval from the university's ethics committee was obtained (decision no: 2022.031.IRB3.003). Institutional approval was also secured from the relevant institution where the study was conducted (decision no: HHD14022022). All participants provided written informed consent after being thoroughly briefed on the study's purpose. The use of the scales in the research was authorized by the original authors through email correspondence. Researchers informed participants of their right to withdraw from the study at any time, with the assurance that data from those who withdrew would not be included in the final analysis. Upon completing the post‐test, participants who completed both the pre and post‐tests and fully participated in the educational program were rewarded with gift vouchers. After completing the RCT, the NGNs in the control group were also granted access to the educational program's online modules.

### Data collection

#### Pilot study

The researchers conducted a pilot study with five nurses who were not part of the study to assess the usability of educational program and the data collection instruments. The questionnaires were revised accordingly based on the feedback received from these participants. These participants were excluded from the final study sample. These revisions pertain to the items in the education evaluation form aimed at assessing participants' views on the online education part. All participants involved in the pilot study suggested that the education evaluation form should consist of two separate sections, with questions allowing for the independent evaluation of the online and face‐to‐face education parts.

#### RCT implementation

RCT was conducted to assess the efficacy of an educational program designed to enhance the empowerment of NGNs, grounded in the SE and PE theories and incorporating the flipped classroom model (Data S3).

#### Implementation steps of the intervention group

- The Descriptive Information Questionnaire, CWEQ‐II, and PES were conducted on the intervention group before starting the education program.
- The mobile‐friendly online education part was implemented on Koç University Online Program Platforms for two weeks. The invitation link to online education was sent to the participants via email. The participants completed the eight modules' educational videos, content, and end‐of‐module practice exercises. In addition, they could share their opinions, thoughts, and questions about education with the educators in the forum section at the end of each module. After the education, the first part of the Education Evaluation Form was applied through the online platform.
- The face‐to‐face education was given to the nurses as a group education for two days in a classroom in the institution where the researchers worked one week after the online education. In the face‐to‐face education part, the participants were involved in the in‐class activities related to the topics included in the online part. After the second day, the second section of the Education Evaluation Form was administered to the intervention group following the face‐to‐face educational session. The CWEQ‐II and PES were conducted again three months later. To assess the long‐term sustainability of any positive outcomes, it is customary in psychosocial intervention studies to conduct a follow‐up evaluation three months postintervention (Breitbart et al., 2015).

#### Implementation steps of the control group

- The Descriptive Information Questionnaire, CWEQ‐II, and PES were conducted on the control group before starting the education program.
- The NGNs in the control group continued to participate in the routine practices of the hospital.
- The CWEQ‐II and PES were conducted again three months later.

### Data analysis

The data were analyzed using SPSS 28 (Statistical Package for the Social Sciences). The statistical analyst was blinded during the data analysis process, and the intervention and control groups were not identified in the data set during coding. Initially, demographic‐ and work‐related characteristics of participants were examined using frequency, percentage, mean, and standard deviation (SD). Next, the demographic‐ and work‐related characteristics, along with CWEQ‐II and PES scores, of both the intervention and control groups were compared. The homogeneity of the outcome variables was assessed using chi‐squared, Fisher's exact, and independent t‐tests. Finally, to evaluate the effects of the educational program, within‐group changes in the CWEQ‐II and PES scores were analyzed using a *t*‐test, and between‐group differences in pretest and posttest results were compared with an independent *t*‐test. A significance level of 0.05 (*p* < 0.05) was applied.

## RESULTS

### Results of homogeneity analysis for demographic‐ and work‐related characteristics and dependent variables

NGNs were randomly allocated to either the intervention group (*n* = 32) or the control group (*n* = 32). There was no sample loss in the pre and post‐tests. The study was ultimately completed with 64 NGNs, comprising 32 individuals in the intervention group and 32 in the control group. At baseline, the demographic‐ and work‐related characteristics exhibited homogeneity between the intervention and control groups. Most participants were female (78.1%) and single (87.5%). The mean age of the participants was 22.84 ± 0.761, and most were 23 years old (40.6%). The mean professional experience was 6.13 months ±1.47, and most had 6 months or more experience (62.5%). Most participants worked in inpatient units (64.1%) and generally worked 48 h/week (84.4%). In addition, all the nurses worked 12 hour shifts, and none had participated in any training on empowerment (Table 2). All participants' initial CWEQ‐II and PES scores were at a moderate level. The intervention group's CWEQ‐II score was 52.53 ± 5.62; the PES score was 33.47 ± 4.34. The control group's CWEQ‐II score was 50.91 ± 7.37; the PES score was 33.25 ± 3.86. The absence of significant differences in the dependent variables of SE and PE suggests homogeneity between the two groups (Tables 3 and 4).

**TABLE 2:** Comparison of the characteristics of NGNs between groups (*n*: 64).

Variables	Intervention (*n*: 32)		Control (*n*: 32)		Total (*n*: 64)		Statistical analysis
	*n*	%	n	%	*n*	%	
Gender							*χ* ^2^: 0.366* *p*: 0.763
Female	26	81.2	24	75	50	78.1	
Male	6	18.8	8	25	14	21.9	
Age: $\bar{x}$ ± SD (22.84 ± 0.761)							*χ* ^2^: 0.321** *p*: 0.852
22 years	11	34.4	13	40.6	24	37.5	
23 years	14	43.8	12	37.5	26	40.6	
24 years	7	21.8	7	21.9	14	21.9	
Marital status							*χ* ^2^: 0.000** *p*:1000
Married	4	12.5	4	12.5	8	12.5	
Single	28	87.5	28	87.5	56	87.5	
Unit type							*χ* ^2^: 0.000* *p*: 1000
In‐patient units	22	68.8	19	59.4	41	64.1	
Intensive care units	5	15.6	6	18.8	11	17.2	
Out‐patient units	1	3.1	2	6.2	3	4.7	
Emergency room	4	12.5	5	15.6	9	14.1	
Experiences $\bar{x}$ ± SD (6.13 ± 1.47)							*χ* ^2^: 0.755** *p*: 0.860
<6 months	12	37.5	12	37.5	24	37.5	
≥6 months	20	62.5	20	62.5	40	62.5	
Weekly working hours							*χ* ^2^: 0.474** *p*: 0.732
48 h/week	26	81.2	28	87.5	54	84.4	
Over 48 h/week	6	18.8	4	12.5	10	15.6	

*Note*: *Fisher's chi‐squared test was used. **Pearson chi‐squared test was used.

Abbreviations: SD, standart deviations; $\bar{x}$, mean.

**TABLE 3:** Intragroup and intergroup comparison of the CWEQ‐II scores in the pre‐ and post‐tests of NGNs (*n*: 64).

Variables	Pretest		Posttest		Statistical analysis	
	$\bar{x}$ ± SD	Min–max	$\bar{x}$ ± SD	Min–max		
CWEQ‐II Total Score						
Intervention (*n*: 32)	52.53 ± 5.62	39–62	67.81 ± 4.07	61–77	t^a^: 23.30	*p* ^a^: <0.001
Control (*n*: 32)	50.91 ± 7.37	38–70	56.22 ± 7.15	43–77	t^a^: 12.58	*p* ^a^: <0.001
Statistical analysis	t^b^: 0.991 *p* ^b^: 0.325		t^b^: 7.96 *p* ^b^: <0.001			
Opportunities						
Intervention (*n*: 32)	8.94 ± 1.70	5–13	11.19 ± 1.40	9–14	t^a^: 9.30	*p* ^a^: <0.001
Control (*n*: 32)	8.88 ± 1.77	5–12	10.03 ± 1.53	7–13	t^a^: 7.40	*p* ^a^: <0.001
Statistical analysis	t^b^: 0.144 *p* ^b^: 0.325		t^b^: 3.14 *p* ^b^: 0.003			
Information						
Intervention (*n*: 32)	8.16 ± 1.32	6–12	11.31 ± 1.40	9–15	t^a^: 12.60	*p* ^a^: <0.001
Control (*n*: 32)	7.38 ± 1.77	4–11	8.53 ± 1.95	6–13	t^a^: 6.41	*p* ^a^: <0.001
Statistical analysis	t^b^: 1.99 *p* ^b^: 0.051		t^b^: 6.55 *p* ^b^: <0.001			
Support						
Intervention (*n*: 32)	9.44 ± 1.24	7–12	11.78 ± 1.26	9–14	t^a^: 10.50	*p* ^a^: <0.001
Control (*n*: 32)	8.34 ± 1.42	6–13	9.19 ± 1.44	7–14	t^a^: 4.83	*p* ^a^: <0.001
Statistical analysis	t^b^: 3.26 *p* ^b^: 0.002		t^b^: 7.64 *p* ^b^: <0.001			
Resources						
Intervention (*n*: 32)	7.63 ± 1.28	6–10	9.53 ± 1.16	7–12	t^a^: 10.19	*p* ^a^: <0.001
Control (*n*: 32)	7.75 ± 1.68	5–11	7.97 ± 1.95	5–13	t^a^: 0.90	*p* ^a^: 0.370
Statistical analysis	t^b^: −0.333 *p* ^b^: 0.740		t^b^: 3.87 *p* ^b^: <0.001			
Formal power						
Intervention (*n*: 32)	6.81 ± 1.42	4–9	9.22 ± 1.15	7–11	t^a^: 13.05	*p* ^a^: <0.001
Control (*n*: 32)	6.84 ± 1.79	3–10	8.22 ± 1.75	5–11	t^a^: 8.25	*p* ^a^: <0.001
Statistical analysis	t^b^: −0.077 *p* ^b^: 0.939		t^b^: 2.69 *p* ^b^: 0.009			
Informal power						
Intervention (*n*: 32)	11.56 ± 1.91	8–15	14.78 ± 1.49	11–18	t^a^:13.61	*p* ^a^: <0.001
Control (*n*: 32)	11.72 ± 1.97	7–15	12.28 ± 1.95	8–16	t^a^: 2.61	*p* ^a^: 0.014
Statistical analysis	t^b^: −0.321 *p* ^b^: 0.749		t^b^: 5.74 *p* ^b^: <0.001			
General empowerment						
Intervention (*n*: 32)	5.84 ± 1.37	3–8	7.50 ± 1.04	5–9	t^a^: 7.59	*p* ^a^: <0.001
Control (*n*: 32)	5.47 ± 1.36	3–8	6.63 ± 1.07	4–9	t^a^: 8.53	*p* ^a^: <0.001
Statistical analysis	t^b^: 1.096 *p* ^b^: 0.277		t^b^: 3.30 *p* ^b^: 0.002			

*Note*: *p* < 0.05.

Abbreviations: CWEQ‐II: Nursing Structural Empowerment Scale; Max, maximum; Min, minimum; SD, standard deviation; $\bar{x}$, mean.

^a^ In‐group evaluation.

^b^ Intergroup evaluation.

**TABLE 4:** Intragroup and intergroup comparison of the PES scores in the pre‐ and posttests of NGNs (*n*: 64).

Variables	Pretest		Posttest		Statistical analysis	
	$\bar{x}$ ± SD	Min–max	$\bar{x}$ ± SD	Min–max		
PES Total Score						
Intervention (*n*: 32)	33.47 ± 4.34	25–41	43.09 ± 4.35	35–52	t^a^: 13.48	*p* ^a^: <0.001
Control (*n*:32)	33.25 ± 3.86	26–42	37.38 ± 3.59	31–45	t^a^: 7.13	*p* ^a^: <0.001
Statistical analysis	t^b^: 0.213 *p* ^b^: 0.832		t^b^: 5.73 *p* ^b^: <0.001			
Meaning						
Intervention (*n*: 32)	10.28 ± 1.68	7–13	12.53 ± 1.70	9–15	t^a^: 11.20	*p* ^a^: <0.001
Control (*n*: 32)	9.84 ± 1.60	7–13	11.38 ± 1.56	9–15	t^a^: 5.53	*p* ^a^: <0.001
Statistical analysis	t^b^: 1.061 *p* ^b^: 0.293		t^b^: 2.83 *p* ^b^: 0.006			
Competence						
Intervention (*n*: 32)	9.53 ± 1.79	5–13	11.53 ± 1.54	8–14	t^a^: 7.22	*p* ^a^: <0.001
Control (*n*: 32)	9.22 ± 1.71	5–12	10.13 ± 1.33	8–13	t^a^: 3.93	*p* ^a^: <0.001
Statistical analysis	t^b^: 711 *p* ^b^: 0.480		t^b^: 3.89 *p* ^b^: <0.001			
Autonomy						
Intervention (*n*: 32)	6.75 ± 2.14	4–11	9.03 ± 1.55	6–13	t^a^: 7.39	*p* ^a^: <0.001
Control (*n*: 32)	7.06 ± 1.66	4–10	8.03 ± 1.49	6–11	t^a^: 5.31	*p* ^a^: <0.001
Statistical analysis	t^b^: −0.652 *p* ^b^: 0.517		t^b^: 2.62 *p* ^b^: 0.011			
Impact						
Intervention (*n*: 32)	6.91 ± 1.72	4–10	10.00 ± 1.39	8–12	t^a^: 10.23	*p* ^a^: <0.001
Control (*n*: 32)	7.13 ± 1.62	3–10	7.84 ± 1.64	6–12	t^a^: 2.52	*p* ^a^: 0.017
Statistical analysis	t^b^: −0.522 *p* ^b^: 0.604		t^b^: 5.65 *p* ^b^: <0.001			

*Note*: *p* < 0.05.

Abbreviations: Max, maximum; Min, minimum; PES, Psychological Empowerment Scale; SD, standard deviation; $\bar{x}$, mean.

^a^ In‐group evaluation.

^b^ Intergroup evaluation.

### Comparison of the mean scores of CWEQ‐II and PES of the participants in‐group and between groups after applying the education program

Statistical analysis revealed a significant difference between the intervention and control groups regarding the total scores of the CWEQ‐II and PES in the posttest conducted 3 months after the educational program (*p* < 0.05). The intervention group demonstrated higher scores than the control group on the total CWEQ‐II scale, including the subdimensions of information, support, resources, and informal power, as well as on the total PES scale, encompassing the subdimensions of competence and impact (see Tables 3 and 4). Within‐group analysis of the total scores for the CWEQ‐II and PES indicated significant differences between pretest and posttest results across all subdimensions in the intervention group (*p* < 0.05). Additionally, statistically significant increases were observed in the pre and post‐test scores of the CWEQ‐II total scale (including opportunities, information, support, and formal power subdimensions) and the PES total scale (including meaning, competence, and autonomy subdimensions) in the control group. However, the posttest scores of the intervention group for both the CWEQ‐II and PES were higher than those of the control group (see Tables 3 and 4).

### Evaluations of the education program

Nurses in the intervention group evaluated the online and face‐to‐face parts of the education program by scoring from 1 to 5. Accordingly, the scores approaching 5 indicate that the relevant statement is very suitable for that education. The participants evaluated the length (4.28 ± 0.68), content (4.69 ± 0.47), technique and method (4.34 ± 0.60), and place (4.81 ± 0.39) of the face‐to‐face education as adequate and appropriate. Participants determined that face‐to‐face education provided them with new knowledge and skills that they could apply in their institutions (4.41 ± 0.61), affected their professional development positively (4.41 ± 0.56), and increased their interest in empowerment (4.50 ± 0.56). While the participants evaluated the length of the education videos (4.19 ± 0.82), the content of the videos (4.59 ± 0.56), and the sound and image quality of the videos (4.66 ± 0.48) as appropriate and adequate, they evaluated the accessibility of the online education program to the user at a more average level (3.94 ± 0.80) compared to other variables. Participants evaluated that online education provided them with new knowledge and skills that they could apply in their institutions (4.38 ± 0.61), contributed positively to their professional development (4.56 ± 0.50), and increased their interest in empowerment (4.53 ± 0.56) (Table 5).

**TABLE 5:** Evaluations of the intervention group about the education program (*n*: 32).

	Min	Max	$\bar{X}$	SD
Variables related to face‐to‐face education				
Was the length of the program adequate?	3	5	4.28	0.68
Was the content of the education appropriate and sufficient?	4	5	4.69	0.47
Were the education method and technique appropriate in terms of understanding the subject?	3	5	4.34	0.60
Was the place suitable for the education?	4	5	4.81	0.39
Did the education contribute positively to your professional development?	3	5	4.41	0.56
Did the education provide you with new knowledge and skills that you can apply in your organization?	3	5	4.41	0.61
Did the education increase your interest in the subject?	3	5	4.50	0.56
Variables related to online education				
Was the length of the educational videos appropriate?	2	5	4.19	0.82
Was the content of the educational videos appropriate and sufficient?	3	5	4.59	0.56
Was the sound and image quality of the educational videos adequate?	4	5	4.66	0.48
Did the use of the online program allow the user to use it easily?	2	5	3.94	0.80
Did the education contribute positively to your professional development?	4	5	4.56	0.50
Did the education provide you with new knowledge and skills that you can apply in your organization?	3	5	4.38	0.61
Did the education increase your interest in the subject?	3	5	4.53	0.56

Abbreviations: Max, maximum; Min, minimum; SD, standard deviation; $\bar{X}$, mean.

## DISCUSSION

The total CWEQ‐II and PES scores of the NGNs in both the intervention and control groups were moderate in the pretest, and no statistically significant differences were found between the mean scores. Similar to the study's results, it was determined that the structural and psychological empowerment levels of NGNs were moderate in other studies (Karimi et al., 2021; Mansour et al., 2022; Sarıköse & Çelik, 2024). When the mean scores of the subdimensions of CWEQ‐II were examined, no significant difference was found between the access to opportunities, access to information, access to resources, formal and informal power, and general empowerment subdimension scores of the intervention and control groups. It was found that the nurses in the intervention group had higher support subdimension scores. This situation is due to the diversity of the NGNs' support resources in their working environments. When the subdimensions of PES were examined, no significant difference was found between the intervention and control groups' meaning, competence, autonomy, and impact scores.

According to the posttest conducted three months after applying for the education program, the CWEQ‐II total scale scores (information, support, resources, and informal power subdimension scores) and PES total scale scores (competence and impact subdimension scores) of the intervention group were higher than those of the control group. These findings indicate that this study's H1 and H2 hypotheses are confirmed. Previous studies found that training programs based on empowering nurses increased satisfaction levels and job satisfaction (Babaeipour‐Divshali et al., 2016; Cicolini et al., 2014). In line with these findings, the education program is effective and increases the empowerment levels of NGNs. This education program is considered to increase the SE perception levels of NGNs by showing how they can access information, support, and resources in their work environments and have practices in terms of experiencing informal power. Özbaş and Tel (2016) found that the burnout levels of nurses decreased, and there was a statistically significant increase in PES scores due to the program they developed for empowering nurses. Empowerment programs with practices that increase the resilience of NGNs and the meaning they attribute to their profession, professional competence, and impact through gaining professional knowledge and skills also increase the PE perceptions of NGNs.

Considering the in‐group changes in the study, in the post‐test conducted three months after the application of the education, a significant increase was seen in the CWEQ‐II and PES total scores and all subdimension scores for intervention groups. This finding shows that this intervention was effective in significantly raising the scores of the NGNs. However, there was a statistically significant increase in the pre and post‐test CWEQ‐II total scores (opportunities, information, support, and formal power subdimension scores) as well as PES total scores (meaning, competence, and autonomy sub‐dimension scores) in the control group three months later. The significant increase in the CWEQ‐II and PES total scores pre and post‐tests in both the intervention and control groups could be explained by the increase in the knowledge, skills, and awareness of all nurses about the working environment and the profession with the increasing working experience and the increase in the professional competence and autonomy levels of NGNs with the decrease in the negative psychological conditions caused by the reality shock. Previous studies showed that nurses' perception of empowerment improved with increasing working experience (Flinkman & Salanterä, 2015; Kuokkanen et al., 2016; Mansour et al., 2022).

NGNs evaluated that this education program contributed positively to their professional development; they gained new knowledge and skills that they could apply in their institutions, and their interest in empowerment increased significantly. Besides, they also stated that they were psychologically empowered and felt more valuable thanks to the education program developed specifically for them. Bard et al. (2022) reported that 96.3% of the nurses stated that the training program added value to their professional development and that they were empowered regarding leadership and professional competencies.

### Limitations

This study was conducted with NGNs employed at a private university hospital, and the sample was restricted to a single institution. Consequently, the generalizability of the findings is limited, as they are influenced by the specific organizational environment and contextual factors of the hospital. The data were collected using self‐reported instruments, specifically the CWEQ‐II and PES, which introduces a potential limitation. Self‐report bias may affect the accuracy of the participants' responses, as individuals may unintentionally provide distorted or subjective accounts of their own behaviors and experiences. The CWEQ‐II and PES (total and most subscales) scores increased significantly in the intervention group as well as in the control group three months later. Work experience may have been a factor in raising the scale scores of NGNs in both groups, a factor that may not have been taken under consideration when designing the study. Therefore, unexplained extraneous variance that may threaten the internal validity of the study is another limitation of this study.

## CONCLUSION

This study showed that the mobile‐friendly and theory‐based education program developed to empower NGNs was an effective intervention that increased their SE and PE levels. According to the study results, since the education program was developed based on empowerment theories, the NGNs accepted its content and components as a well‐structured education program to meet the objectives of the education, and the flipped classroom model was evaluated as a comfortable, educational, interesting, and effective education for adult education. However, the content of the online part of the education program that will be more user‐friendly may increase participants' satisfaction.

According to other studies, nurse residency and orientation programs have demonstrated positive outcomes, benefiting NGNs through education, support, and guidance (Murray et al., 2020; Van Camp & Chappy, 2017). Evidence indicates that NGNs participating in these programs are better prepared to navigate the challenges of nursing in today's complex healthcare environment. However, these programs often lack a specific theoretical framework and practices aimed at empowering new graduates. The education program we developed to empower NGNs, identified as an effective intervention, is recommended to be integrated into nurse residency and orientation programs across healthcare organizations. Additionally, future research should apply this education program to a more extensive and diverse sample, encompassing multiple institutions. There is a need to carry out studies and activities to disseminate this program. The findings of this study will guide educators, researchers, and administrators in future strategies and innovative programs for empowering NGNs.

### Clinical Resource

- American Nurses Association (ANA): Practice Ready Nurse Graduates|RN Initiative|Foundation (nursingworld.org).
- International Council of Nurses (ICN): Canadian Nurses Association and the International Council of Nurses co‐host “Nursing. Power. Advocacy—Helping Nurses Deliver”|ICN—International Council of Nurses.
- Sigma Theta Tau International Honor Society of Nursing (Sigma): Sigma Nursing with Johnson & Johnson Foundation launch 2023 Nurse Empowerment Program for frontline nurses.

## FUNDING INFORMATION

This research was obtained from the project numbered 122K327, supported by TÜBİTAK 1001—The Scientific and Technological Research Projects Funding Program and derived from Seda Sarıköse's doctoral thesis.

## CONFLICT OF INTEREST STATEMENT

The authors declare no conflicts of interest.

## Supporting information


**Data S1.** Supporting Information.

**Data S2.** Supporting Information.

**Data S3.** Supporting Information.

## Data Availability

The data that support the findings of this study are available upon request from the corresponding author. The data are not publicly available due to privacy or ethical restrictions.
