# Supplementary figures and images for: Evaluating the effectiveness of the education program developed for the empowerment of new graduate nurses: A randomized controlled trial
Source: J Nurs Scholarsh. 2024 Dec 10;57(3):514–26. doi: 10.1111/jnu.13041 (PMC12064837; doi:10.1111/jnu.13041)

Supplemental Content 2. Images from the Online Part of the Education Programme


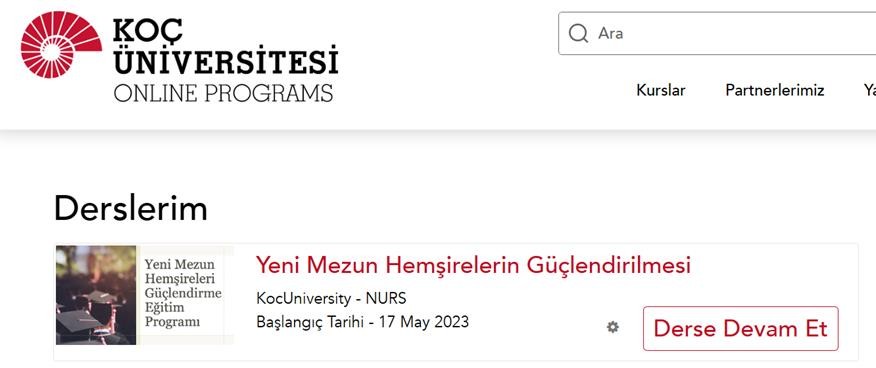

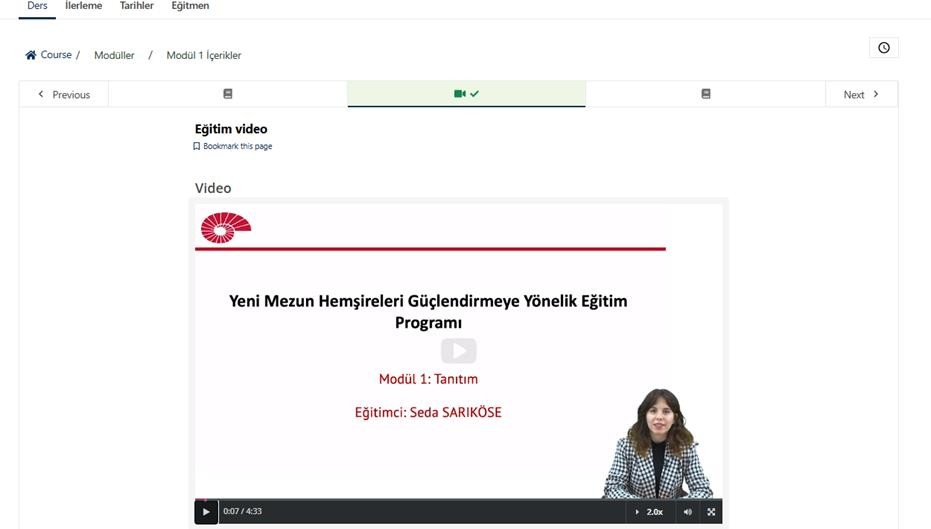

Supplement: Supplementary file 2 — Data S2. Supporting Information. [file JNU-57-514-s003.docx]
