# Supplementary material for: Evaluating the effectiveness of the education program developed for the empowerment of new graduate nurses: A randomized controlled trial
Source: J Nurs Scholarsh. 2024 Dec 10;57(3):514–26. doi: 10.1111/jnu.13041 (PMC12064837; doi:10.1111/jnu.13041)
Supplement: Supplementary file 3 — Data S3. Supporting Information. [file JNU-57-514-s001.docx]

**Supplemental Content 3. Implementation Steps of the Intervention and Control Group**
